# Prognosis and serum creatinine levels in acute renal failure at the time of nephrology consultation: an observational cohort study

**DOI:** 10.1186/1471-2369-8-14

**Published:** 2007-09-26

**Authors:** Jose Ramon Perez-Valdivieso, Maira Bes-Rastrollo, Pablo Monedero, Jokin de Irala, Francisco Javier Lavilla

**Affiliations:** 1 Department of Anesthesia and Critical Care, Clinica Universitaria, University of Navarra, Pamplona, Spain; 2 Department of Preventive Medicine and Public Health, University of Navarra, Pamplona, Spain; 3 Renal Unit, Clinica Universitaria de Navarra, University of Navarra, Pamplona, Spain

## Abstract

**Background:**

The aim of this study is to evaluate the association between acute serum creatinine changes in acute renal failure (ARF), before specialized treatment begins, and in-hospital mortality, recovery of renal function, and overall mortality at 6 months, on an equal degree of ARF severity, using the RIFLE criteria, and comorbid illnesses.

**Methods:**

Prospective cohort study of 1008 consecutive patients who had been diagnosed as having ARF, and had been admitted in an university-affiliated hospital over 10 years. Demographic, clinical information and outcomes were measured. After that, 646 patients who had presented enough increment in serum creatinine to qualify for the RIFLE criteria were included for subsequent analysis. The population was divided into two groups using the median serum creatinine change (101%) as the cut-off value. Multivariate non-conditional logistic and linear regression models were used.

**Results:**

A ≥ 101% increment of creatinine respect to its baseline before nephrology consultation was associated with significant increase of in-hospital mortality (35.6% vs. 22.6%, p < 0.001), with an adjusted odds ratio of 1.81 (95% CI: 1.08–3.03). Patients who required continuous renal replacement therapy in the ≥ 101% increment group presented a higher increase of in-hospital mortality (62.7% vs 46.4%, p = 0.048), with an adjusted odds ratio of 2.66 (95% CI: 1.00–7.21). Patients in the ≥ 101% increment group had a higher mean serum creatinine level with respect to their baseline level (114.72% vs. 37.96%) at hospital discharge. This was an adjusted 48.92% (95% CI: 13.05–84.79) more serum creatinine than in the < 101% increment group.

**Conclusion:**

In this cohort, patients who had presented an increment in serum level of creatinine of ≥ 101% with respect to basal values, at the time of nephrology consultation, had increased mortality rates and were discharged from hospital with a more deteriorated renal function than those with similar Liano scoring and the same RIFLE classes, but with a < 101% increment. This finding may provide more information about the factors involved in the prognosis of ARF. Furthermore, the calculation of relative serum creatinine increase could be used as a practical tool to identify those patients at risk, and that would benefit from an intensive therapy.

## Background

Acute renal failure (ARF) is a life threatening illness with a high mortality despite advances in supportive care [1-14]. Although there is a strong and direct relation between multiorgan failure and in-hospital mortality, severity of illness does not explain the variation in outcomes among patients with acute renal failure [5,9,10]. There is an increased cost in terms of patient prognosis, financial and clinical management. In a study, 30% of patients did not recover completely their renal function [15] and, in other studies, progression to chronic renal failure in many patients is suggested [9,16]. In regard to these concerns, prompt recognition and early consultation with a nephrologist have been postulated to improve the outcome of patients with acute renal failure [7,11,12,17]. Nevertheless, the published evidence is scarce.

The absence of a consensus definition of ARF has made research about ARF difficult [6,18,19]. Recently, the Acute Dialysis Quality Initiative (ADQI) published a uniformed definition called the RIFLE (Risk, Injury, Failure, Loss, End Stage) criteria [20]. The RIFLE criteria has been adopted by the Acute Kidney Injury Network (AKIN) [6].

Diagnosis of ARF based upon changes in serum creatinine may be delayed due to the fact that, in the non-steady-state conditions of ARF, as GFR falls creatinine secretion is increased [11]. Large changes in GFR are initially manifested as small quantitative changes in serum creatinine in the first 24–48 hours after renal injury [11]. After these one or two days, the degree of serum creatinine changes will reflect the change in GFR. Finally, the serum creatinine is stabilized, and that takes about 7 days [11,20]. Therefore, it is almost impossible to exactly determine the onset of the ARF; and also calculating the exact time lapsed until the nephrology consultation. However, measuring serum creatinine level is a practical approach for discovering short-term alteration in renal function, despite its limitations, because it is readily used in clinical practice and it is specific for renal function [20,21]. In patients with stable renal function, those levels are constant, with a daily variability of 8% [22,23], so increasing levels might suggest the first stage of ARF.

We conducted this study to identify and quantify a correlation between acute serum creatinine changes in ARF, measuring them at the time of nephrology consultation, and mortality and recovery of renal function. We hypothesized that, at the same levels of severity of renal and comorbid illnesses, the higher the acute serum creatinine reached with respect to its baseline, before the nephrologist first began to treat the patient, the worse the outcome will be.

## Methods

### Study population

The University Hospital of Navarra is a tertiary care academic teaching medical center with 400 beds in the city of Pamplona, Spain. After hospital ethics committe approval, a cohort of 1008 consecutive patients who had been diagnosed as having acute renal failure, and had been admitted in our hospital between 1996 and 2006, was prospectively entered in a computerized database. Explicit patient consent was not required by the hospital ethics committee due to the observational nature of the study, and because collecting the data was a part of our academical-hospital routine work. Patients' anonymity was always strictly preserved. We restricted our analysis to patients who had enough increase of serum creatinine during their admission to fulfill the RIFLE criteria for ARF [20], and were older than 16 years. Three hundred and six patients did not fulfill the RIFLE serum creatinine criteria nor presented oliguria during their stay. Forty-nine patients presented oliguria, but did not have substantial increase of creatinine. Seven patients were younger than 16 years. Finally, 646 patients were included for subsequent analysis.

### Definition of the variables

ARF was defined and diagnosed if patients had showed substantial increment in serum creatinine during their admission to qualify for the RIFLE criteria. After that, patients were classified to the maximum RIFLE class according to the peak creatinine (the highest) reached during their hospital stay. On the contrary, we could not collect full information for urine output, so we were not able to classify the patients by that RIFLE criteria. For patients without chronic renal failure reported, the baseline creatinine was calculated using the Modification of Diet in Renal Disease (MDRD) equation [24], as recommended by the ADQI workgroup [20], assuming a glomerular filtration rate of 75 ml/minute/1,73 m^2^. For patients with a history of renal failure the baseline creatinine was defined as the one measured at hospital admission [4]. The term community-acquired ARF was used when the patient had presented ARF on admission to the hospital.

Demographic data, etiology of acute renal failure, comorbid conditions, severity of illness, and laboratory data were all prospectively collected. The difference between serum creatinine value at time when nephrologist first saw the case and baseline value was calculated and expressed as a percentage: Creatinine change (%) = [(creatinine when nephrologist saw the case - basal creatinine)/basal creatinine] * 100. Severity of illness was measured through Liano score (0.032*age in decades - 0.086*male gender - 0.109*nephrotoxic + 0.109*oliguria + 0.116*hypotension + 0.122*jaundice + 0.150*coma - 0.154*consciousness + 0.182*assisted respiration+ 0.210) [21], Karnofsky score at home [25], and prior food intake were also determined and computed at that point. The patient's clinical status and treatment ARF were recorded daily. Serum creatinine at hospital discharge was recorded as a measure of recovery of renal function; the difference between this value and the baseline value was calculated and expressed in percentage: Creatinine change (%) = [(creatinine at hospital discharge - basal creatinine)/basal creatinine] * 100. Food intake was defined as previous caloric ingestion, and this was classified as appropriate when it was optimal, light malnutrition when it had been inappropriate less than three days, moderate malnutrition when it had been inappropriate between three and seven days, and severe malnutrition when it had been inappropriate for more than seven days. We followed up the patients after they were discharged from hospital to study the mortality at 6 months from the beginning of nephrology consultation.

Serum creatinine concentration was measured using the kinetic Jaffe assay.

In our hospital, when renal function is impaired, nephrology consulation can be requested, no matter in which hospital ward the patient stays. A nephrologist is always the only physician who provides a renal replacement therapy, according to a strict protocol. However, opinion from other medical departments involved in the patient's care is always sought. In this study, all patients were treated after the consultation by the same nephrologist, and all the data were gathered by the same observer.

The primary endpoints were in-hospital mortality and recovery of renal function at hospital discharge, following the recommendations from the ADQI [26]. The secondary endpoint was overall mortality at 6 months.

### Statistical analysis

The increase in the percentage of creatinine reached before the nephrologist first saw the case was considered as the main exposure variable. The patients were divided into two groups using the median (101%) as the cut-off value. In addition, this cut-off point was the most optimal point from a ROC analysis. We obtained one below the median (group 1 or < 101% increment, hereafter), and one above the median (group 2 or ≥ 101% increment, hereafter). Non-conditional logistic regression models were fitted to assess the relationship between the increase of percentage in creatinine and the risk of in-hospital mortality. Odds ratios (OR) and their 95% confidence intervals (CI) were calculated considering the < 101% increment group as the reference category. The OR represents an estimation of relative risks of death during the inpatient stay.

In addition, linear regression models were used to assess the association between the increment of creatinine at time when nephrologists saw the case (exposure), and the recovery of renal function considering the increase in the percentage of creatinine at hospital discharge with respect to the baseline values (outcome). After excluding inhospital dead patients, we estimated the regression coefficient and their 95% CI for the larger increment group compared to the lesser increment group considered as the reference category. This coefficient represents the absolute difference in creatinine increment at hospital discharge between the ≥ 101% increment group and the < 101% increment group.

In both non-conditional logistic regression and linear regression, we fitted a crude model (univariate, i.e., without any adjustment), an age and gender model, and a multivariate-adjusted model including the following variables: Liano scoring, Karnofsky scoring, prior food intake, chronic renal failure, diabetes, treatment of acute renal failure, causes of acute renal failures, community-acquired acute renal failure, basal hemoglobin, basal serum albumin, and RIFLE classes selected by the descriptive univariate analysis of potential confounders with a p value less than 0.10. The Liano scoring includes in its equation the variables for nephrotoxicity, oliguria, hypotension, jaundice, mental status, and assisted respiration. Continuous variables were expressed as medians (and interquartile ranges), and compared using Mann-Whitney U test. Categorical variables were expressed as proportions and compared with the Chi-squared test. We evaluated all first-order multiplicative interactions (effect modifications) through product terms.

Overall survival at 6 months across groups was analyzed using the Kaplan-Meier methods, and differences between groups were tested using the log-rank test. The time of origin was the date when the nephrology consultation started. The event defined was death whereas those cases alive at the end of follow-up and those lost to follow-up were censored at their last observation.

All p values presented are two tailed, p < 0.05 was considered statistically significant. Statistical analyses were conducted using SPSS v.12.0.1 (SPSS Inc., Chicago, IL, USA).

## Results

Six hundred and forty-six patients were evaluated, among them one hundred and eighty-eight died in-hospital. The demographics and baseline clinical characteristics are shown in Table 1. Subjects in the ≥ 101% increment of creatinine group were more likely to be female (p = 0.01), had impaired prior food intake (p = 0.02), and required more renal replacement therapy (p < 0.001). The ≥ 101% increment of creatinine group was associated with high prevalence of community-acquired ARF (p = 0.02), and worse Karnofsky scoring (p = 0.015). The group with ≥ 101% increment of creatinine was also associated with higher Liano scoring at the time of nephrology consultation (p < 0.001), including higher prevalence of hypotension (p < 0.001), oliguria (p < 0.001), jaundice (p = 0.008), coma (p = 0.027), and were on mechanical ventilation (p = 0.002). RIFLE classes reached during the ARF were more severe in subjects in the ≥ 101% increment of creatinine group (p < 0.001). There was a higher prevalence of diabetes (p = 0.037) in this group.

**Table 1:** Characteristics of patients, according to percentage of difference of creatinine* at nephrology consultation with respect to basal creatinine

	**GROUPS OF % CREATININE INCREASE**		
	**< 101% increment**	**≥ 101% increment**	**p value**

*N of patients*	323	323	
*Increase of Creatinine, median % (IQR)*.	34.07 (66.67)	201.52 (149.22)	
Age, median years (IQR).	63 (20)	62 (17)	0.36
Male gender (%)	74.3	65.0	0.01
Surgical (%)	24.9	24.2	0.849
Oncology patients (%)	36.5	34.4	0.565
Patients with no previous history of CRF (%)	61.30	91.64	< 0.001
Basal Serum Creatinine according to the MDRD	1.04 (0.19)	1.04 (0.22)	0.25
equation, median (mg/dL) (IQR).			
Patients with history of CRF (%)	38.70	8.36	< 0.001
Basal Serum Creatinine for CRF patients, median	2.3 (2.2)	1.5 (0.6)	< 0.001
mg/dL (IQR),			
RIFLE criteria (%)			< 0.001
Risk	35.9	0.0	
Injury	26.6	29.1	
Failure	37.5	70.9	
Basal Serum Albumin, median g/dL (IQR),	2.62 (1.07)	2.42 (0.86)	0.072
Basal Hemoglobin, median g/dL (IQR)	10.70 (3.10)	10.50 (3.20)	0.088
Community-acquired Acute Renal Failure (%)	36.0	46.6	0.020
Liano scoring, median (IQR)	0.23 (0.27)	0.30 (0.44)	< 0.001
Karnofsky scoring, median (IQR)	70 (20)	60 (10)	0.015
Hypotension (%)	30.3	45.5	< 0.001
Oliguria (%)	31.3	50.8	< 0.001
Jaundice (%)	22.0	31.3	0.008
Coma (%)	11.8	18.0	0.027
Conciousness (%)	80.8	76.8	0.211
Mechanical ventilation (%)	18.3	28.5	0.002
Aminoglycoside use (%)	12.7	17	0.121
Radiocontrast procedures (%)	19.5	20.7	0.695
Diabetic (%)	11.1	6.5	0.037
Nephrotoxicity (%)	43.0	43.7	0.874
Causes of Acute Renal Failure			0.079
Pre-renal (%)	77.1	72.1	
Intrinsic renal (%)	18.9	19.2	
Post-renal (%)	2.2	3.4	
Other causes (%)	1.9	5.3	
Subsecuent Treatment of Acute Renal Failure			< 0.001
Non-Dialytic (%)	75.5	61.0	
Intermittent Hemodialysis (%)	7.1	7.7	
Continuous Replacement Therapy (%)	11.1	22.6	
Both (Intermittent+Continuous) (%)	6.2	8.7	
Prior food intake			0.024
Optimal nutrition (%)	37	24	
Light malnutrition (%)	18.1	20.7	
Moderate malnutrition (%)	23.9	30.9	
Severe malnutrition (%)	21	24.4	

*Creatinine change (%) = [(creatinine at nephrology consultation - Basal creatinine)/Basal creatinine] × 100 IQR: Interquartile range. CRF: Chronic Renal Failure. MDRD: Modification of Diet in Renal Disease

The ≥ 101% increment group was associated with significant increased total inhospital mortality (35.6% vs. 22.6%, P < 0.001), with an adjusted odds ratio of 1.81 (95% CI: 1.08–3.03) (Table 2). Moreover, those patients in the ≥ 101% increment group who required continuous renal replacement therapy presented a higher increase of inhospital mortality (62.7% vs 46.4%, P = 0.048), with an adjusted odds ratio of 2.66 (95% CI: 1.00–7.21) (Table 3). We noticed that patients in the ≥ 101% increment group had a higher mean serum creatinine level in comparison to their baseline level (114.72% vs. 37.96%) at hospital discharge (Table 4). In the ≥ 101% increment group, creatinine was an adjusted 48.92% (95% CI: 13.05–84.79) higher than the < 101% increment group.

**Table 2:** Odds Ratios (ORs) and (95% confidence intervals) for in-hospital mortality; according to the increased percentage of creatinine with respect to basal creatinine*

	**GROUPS OF % CREATININE INCREASE**		
	**< 101% increment**	**≥ 101% increment**	**p value**

Creatinine and mortality			
*n*	323	323	
Incidence of in-hospital mortality, n (%)	73 (22.6)	115 (35.6)	

Odds Ratios			

Crude OR (95% CI)	1.00 (Ref.)	1.89 (1.34–2.68)	< 0,001
Age- and sex-adjusted OR (95% CI)	1.00 (Ref.)	1.91 (1.35–2.70)	< 0,001
Multivariate-adjusted OR^† ^(95% CI)	1.00 (Ref.)	1.81 (1.08–3.03)	0.024
Additionally adjusted for Oncology patients	1.00 (Ref.)	1.85 (1.09–3.14)	0.023

* Creatinine change (%) = [(creatinine at nephrology consultation - Basal creatinine)/Basal creatinine] × 100

^†^Adjusted for age, sex, Liano scoring, Karnofsky scoring, prior food intake, chronic renal failure, diabetes, treatment of acute renal failure, causes of acute renal failure, community-acquired acute renal failure, basal hemoglobin, basal serum albumin, and RIFLE classes.

**Table 3:** Odds Ratio (ORs) and (95 % confidence intervals) for in-hospital mortality; according to the increased percentage of creatinine* with respect to basal creatinine in patients who required Continuous Renal Replacement Therapy subsecuently

	**GROUPS OF % CREATININE INCREASE**		
	**< 101% increment**	**≥ 101% increment**	**p value**

Creatinine and mortality			
*n*	56	102	
Incidence of in-hospital mortality, n (%)	26 (46.4)	64 (62.7)	

Odds Ratios			

Crude OR (95% CI)	1.00 (Ref.)	1.94 (1.00 – 3.76)	0.048
Age- and sex-adjusted OR (95% CI)	1.00 (Ref.)	2.01 (1.03 – 3.92)	0.040
Multivariate-adjusted OR^† ^(95% CI)	1.00 (Ref.)	2.66 (1.00 – 7.21)	0.050
Additionally adjusted for Oncology patients	1.00 (Ref.)	2.88 (1.03 – 8.09)	0.044

*Creatinine change (%) = [(creatinine at nephrology consultation - Basal creatinine)/Basal creatinine] × 100

^†^Adjusted for age, sex, Liano scoring, Karnofsky scoring, prior food intake, chronic renal failure, diabetes, treatment of acute renal failure, causes of acute renal failure, community-acquired acute renal failure, basal hemoglobin, basal serum albumin, and RIFLE classes.

**Table 4:** Estimates (regression coefficients and 95% confidence intervals) for the subsequent outcome in % creatinine* (mg/dL) at discharge from hospital according to increased percentage of creatinine ^† ^with respect to basal creatinine

	**GROUPS OF % CREATININE INCREASE**		
	**< 101% increment**	**≥ 101% increment**	**p value**

Creatinine			
*n*	250	208	
Absolute Creatinine change*	+37.96	+114.72	
[%, mean (95% CI)]	(29.46–46.47)	(83.79–145.65)	

Differences in Creatinine change			

Crude (Regression coefficient, β)	0 (Ref.)	+76.76 (+47.14 to +106.38)	< 0.001
Age- sex-adjusted	0 (Ref.)	+71.27 (+41.78 to +100.77)	< 0.001
(Regression coefficient, β)			
Multivariate adjusted model^‡^	0 (Ref.)	+48.92 (+13.05 to +84.79)	0.008
(Regression coefficient, β)			
Additionally adjusted for	0 (Ref.)	+49.93 (+14.02 to +85.83)	0.007
Oncology patients			

*Creatinine change (%) = [(creatinine at nephrology discharge - Basal creatinine)/Basal creatinine] × 100

^†^Creatinine change (%) = [(creatinine at hospital consultation - Basal creatinine)/Basal creatinine] × 100

^‡^Adjusted for age, sex, Liano scoring, Karnofsky scoring, prior food intake, chronic renal failure, diabetes, treatment of acute renal failure, causes of acute renal failure, community-acquired acute renal failure, basal hemoglobin, basal serum albumin, and RIFLE classes.

**Figure 1:**
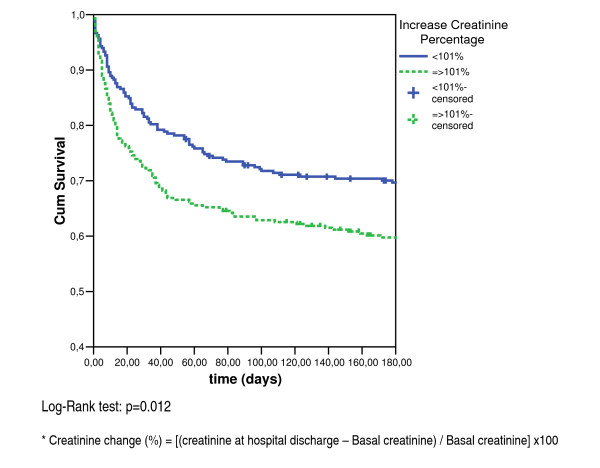
Six-month survival after starting the nephrology consultation according to increase creatinine percentage*.

The overall mortality rate was 337 deaths (52.2%) in 6 months. According to the Kaplan-Meier plot, the ≥ 101% increment group also had a statistically significant higher mortality rate in this period as compared with the < 101% increment group (Log Rank test = 0.012) (Figure 1). During the first 60 days of follow-up 175 deaths (27.1%) occurred.

## Discussion

This longitudinal study shows that a relative increase in baseline serum creatinine by ≥ 101% compared with the levels measured at the time of nephrology consultation is an independent predictor of mortality. Such an increase was also associated with worse functional renal recovery irrespective of the degree of ARF severity or presence of comorbid illnesses.

Due to the heterogeneous nature of ARF [11], it was important to adjust for the severity of illness with Liano score, and for the acute kidney injury with the RIFLE criteria. We have chosen the Liano score because of its ability to discriminate mortality from survival and its ability to calibrate the observed mortality rate with the expected mortality in ARF [11,14,27]. The RIFLE consensus criteria gives a standard definition and a level of classification of severity in ARF [20], a request often made by the experts [6,18,19]. Working in this manner may allow continuing further investigation in ARF, and is one of the main strengths of this study. Patients did not do worse because they had more severe ARF or comorbid illnesses. Instead, patients presenting the same RIFLE criteria and Liano scores, but with higher serum creatinine increments prior to the nephrology consultation, showed higher mortality rates.

Using multivariate logistic regression analysis, we found that an increment in creatinine with respect to baseline level of 101% at the time of nephrology consultation started is a factor independently and strongly predictive of death in this cohort. Doubling the basal creatinine level presented an odds ratio of 1.81 for mortality among general population. This increased to a threefold (OR = 2.66) mortality in patients who needed continuous renal replacement therapy. These results might suggest that the increase of serum creatinine level at the moment when the ARF began to be treated by an expert was related to the outcome. However, we should concede the possibility that the more abrupt increase in serum creatinine was, the worse the prognosis. Because of the observational nature of the present study, our findings need to be interpreted with caution. We cannot determine with certainty that finding a higher increase in the percentage of creatinine was a consequence of a delayed nephrology consultation. It could be that among patients who received the nephrology intervention at the same time, some of them presented a higher increase in the percentage of creatinine because of an abrupt deterioration of renal function. Nevertheless, after adjusting for severity of ARF and comorbid illnesses, patients who had had higher increments in percentage of creatinine prior to being treated by a nephrologist did present higher mortality rates. We might stand for prompt expert consultation because these ARF patients at risk would benefit from an intensive and specialized treatment, and further kidney damage could be minimized.

The cohort included a heterogeneous distribution of patients, in terms of unit, nature, or source of admission; but with an important number of oncology patients (35.4%) since our institution is a well-known center in Spain for the treatment of oncologic diseases. For this reason, we adjusted adding the oncology factor to the logistic regression in an extra analysis. However, because of its longitudinal design, we could obviate the Berkson bias (to take a falsely typical population of patients since the group of people being studied has no form of control over whether to participate) [28]. Also, it could be argued that this was a single center study, with its limits to apply to other hospitals. We have tried to minimize any bias using objetive data collected by a single investigator following strict criteria, and reviewing the data. Urine output data were not available; therefore it was not possible to estimate the RIFLE criteria according to this value. We started to collect the data before the RIFLE criteria was formulated, and most of the ward patients had no urine output measured on a six hourly basis. We assume that we might underestimate some cases according to the RIFLE criteria; but only 21 and 62 patients from groups R and I respectively had oliguria. Although we cannot rule out the possibility of residual confounding, it is unlikely that it can fully explain the observed strong associations. Moreover, when we adjusted for RIFLE criteria, an increase of our estimations occurred in the multivariate model; so misclassification of this covariate would most likely bias the odds ratios toward the null value.

We calculated the MDRD equation to obtain a baseline serum creatinine in patients with no previous history of renal failure because a true baseline is often unknown. We solved the equation assuming a GFR of 75 ml/minute/1,73 m^2^, which has been reported to estimate a lower limit of a normal GFR [4,13,20]. Because GFR was assumed to be 75 ml/min, using MDRD for patients with unknown pre-morbid renal dysfunction may underestimate severity of ARF according to the RIFLE criteria [29,30]. This lack of reliability is one of the controversial issues regarding the RIFLE criteria, but it is still unresolved [31]. Although the MDRD formula may be less accurate at normal GFR, resulting in overdiagnosis of ARF, it should not be a disadvantage in a clinical setting where ARF is taken seriously, and the MDRD also has the advantage of not requiring body weight data [32] that could be wrongly estimated in fluid retention settings.

We chose to calculate the percentage change in creatinine respect to its baseline because serum creatinine is modulated by muscle bulk. Therefore, smaller changes as 0.3 or 0.5 mg/dL will have distinct significance for patients with different genders and/or ages, whose normal baseline levels are different [18]. For this reason, we found that a relative increase in creatinine was more accurate than an absolute increase one.

The higher presence of chronic renal failure in the < 101% increment group might be explained because concern about it could have induced earlier nephrology consultation, even if the patient did not present signs of ARF in that time. However, we must admit that 74 chronic renal failure patients (48.68%) might have presented an unstable serum creatinine value at hospital admission, thus underestimating the severity of ARF for them. Chronic renal failure was included in this study to represent the common clinical practice, and thereafter it was adjusted for in the analysis because of expecting different outcomes than the general population.

On the other hand, the higher proportion of cases of ARF developed in the community in the ≥ 101% increase group might suggest that nephrology consultation may have been delayed because of time spent before hospital admittance, giving serum creatinine an extra time to reach a higher value.

Our results about mortality in the Renal Replacement Therapy sub-group are consistent with previously reported [9,33,34], and it is of interest the higher mortality rate in patients in the ≥ 101% increase group. What we may learn here is that this group of patients is most likely to benefit from a more intensive treatment.

The degree of renal injury is likely to affect renal recovery. Patients where consultation was started with ≥ 101% creatinine increase had significant higher creatinine levels at discharge compared to their baseline levels, and many of them would be expected to develop chronic renal malfunction. Adjustment for confounding factors in the analysis was done, too.

We analyzed 6-months mortality separately, since the time when patients are discharged from hospital might vary between admission units. Although it could be argued that all-cause mortality at six months is non specific, we found that a high number of deaths (175) occurred in the first 60 days. These findings agree with previous reports and recommendations about optimal follow-up time in patients with established ARF [20,26,35].

It is of interest to note that 306 patients from the original population of 1008 would not have been diagnosed of ARF had the RIFLE criteria been used then. They did not present oliguria nor enough creatinine increment, but were diagnosed of ARF according to another criteria at that time. Some experts might prefer to use more inclusive criteria because of concern about under-recognizing ARF [6].

Finally, had our patients presented other mortality rates, if they would have been treated by a nephrologist before reaching serum creatinine levels more than twice their baseline levels, is a question beyond this study and needs further research.

## Conclusion

At the same degree of severity of kidney injury and comorbid illnesses, while controlling for other potential confounders, ARF patients with a relative increase of ≥ 101% in the baseline creatinine at the time of the nephrology consultation was an independent predictor of mortality and worse prognosis. The calculation of relative serum creatinine increase could be used as a precise and useful tool in the daily general medical practice, and may be an effective tool to identify those patients who would benefit from an intensive therapy. However, further investigation about timing in nephrology consultation is needed to provide robust knowledge about treatment and prognosis in ARF.

## Key messages

1. Acute renal failure is a condition with a high risk of mortality.

2. At the same degree of acute renal failure and comorbid illnesses severity, and receiving similar treatment, patients who had presented an increment of creatinine ≥ 101% with respect to baseline at the time of nephrology consultation showed increased mortality rates.

3. Those patients were discharged from hospital with more deteriorated renal function.

4. The calculation of relative serum creatinine increase might be used as a precise and useful tool in the general medical practice. Predicting the outcome helps to identify those patients who would benefit from a intensive therapy, and also to reassure patients and their relatives.

## Abbreviations

ARF: Acute Renal Failure.

RIFLE: Risk, Injury, Failure, Loss, End Stage Classification for acute kidney injury.

ADQI: Acute Dialysis Quality Initiative.

GFR: Glomerular Filtration Rate.

AKIN: Acute Kidney Injury Network.

MDRD: Modification of Diet in Renal Disease

OR: Odds Ratio.

CI: Confidence Intervals.

## Competing interests

The author(s) declare that they have no competing interests.

## Authors' contributions

All the authors conceived the research question. JRPV and MBR wrote the first draft and conducted the statistical analysis with advice from JdI, PM and JL. PM and JL coordinated the research. JL contributed to data collection. Subsequent revisions were carried by JRPV and MBR with input from all authors. The final version was approved by all authors.

## Pre-publication history

The pre-publication history for this paper can be accessed here:


